# Biochar Nanocomposite as an Inexpensive and Highly Efficient Carbonaceous Adsorbent for Hexavalent Chromium Removal

**DOI:** 10.3390/ma15176055

**Published:** 2022-09-01

**Authors:** Soroosh Mortazavian, Simona E. Hunyadi Murph, Jaeyun Moon

**Affiliations:** 1 Department of Mechanical Engineering, University of Nevada Las Vegas, Las Vegas, NV 89154, USA; 2 Savannah River National Laboratory (SRNL), Aiken, SC 29808, USA

**Keywords:** hexavalent chromium, hydrophilic biochar, biochar modification, cost-effective adsorbent, zero-valent metals, activated carbon substitute

## Abstract

Biochar is commonly used for soil amendment, due to its excellent water-holding capacity. The Cr(VI) contamination of water is a current environmental issue in industrial regions. Here, we evaluated the effects of two-step modifications on boosting biochar’s performance in terms of the removal of aqueous hexavalent chromium (Cr(VI)), along with investigating the alterations to its surface properties. The first modification step was heat treatment under air at 300 °C, producing hydrophilic biochar (HBC). The resulting HBC was then impregnated with zero-valent iron nanoparticles (nZVI), creating an HBC/nZVI composite, adding a chemical reduction capability to the physical sorption mechanism. Unmodified biochar (BC), HBC, and HBC/nZVI were characterized for their physicochemical properties, including surface morphology and elemental composition, by SEM/EDS, while functional groups were ascertained by FTIR and surface charge by zeta potential. Cr(VI) removal kinetic studies revealed the four-time greater sorption capacity of HBC than BC. Although unmodified BC showed faster initial Cr(VI) uptake, it rapidly worsened and started desorption. After nZVI impregnation, the Cr(VI) removal rate of HBC increased by a factor of 10. FTIR analysis of biochars after Cr(VI) adsorption showed the presence of Cr(III) oxide only on the used HBC/nZVI and demonstrated that the carbonyl and carboxyl groups were the main groups involved in Cr(VI) sorption. Modified biochars could be considered an economical substitute for conventional methods.

## 1. Introduction

With population growth, industrialization, and growing water demand, the development of inexpensive, widely available, and effective materials for water purification and wastewater treatment has recently attracted considerable research efforts in the environmental/materials engineering field [1]. Due to the simple operation and a wide range of applications, adsorption has been considered for some time to be the primary and most widely applied method for drinking water purification and wastewater treatment. Activated carbon has been known as the primary choice of adsorbent for water stewardship applications due to its high efficiency in the removal of a wide range of organic and inorganic water contaminants. However, there are a number of drawbacks associated with activated carbon as a water treatment sorbent. This includes its high production cost, slow reaction rate, and difficult regeneration [2,3]. Therefore, there is a great deal of interest in the environmental and materials engineering scientific community in the development of alternative sorbents or the modification of previously developed sorbents to provide specificity for target contaminants while optimizing the cost and efficiency of the process. For example, we recently reported an nZVI-biochar composite used for aqueous trichloroethylene (TCE) removal [2] and activated carbon coated with polysulfide polymer used for hexavalent chromium (Cr(VI)) removal [4]. On the other hand, various composites containing immobilized zero-valent iron nanoparticles (nZVI) have been investigated for Cr(VI) removal, including a composite of copper and nZVI [5], a bentonite-supported organosolv lignin-stabilized nZVI [6], and olive stones coated with nZVI and magnetite nanoparticles [7]. Additionally, sustainable and green synthesis methods have been recently developed for the synthesis of materials for Cr(VI) removal, such as a willow-leaf iron-based composite [8] and a bacterial cellulose/polyaniline nanocomposite aerogel [9]. 

Biochar is commonly used for soil amendment due to its excellent water-holding capacity, porous structure, and the presence of abundant organic matter, making it an environmentally friendly resource [10]. For example, Su et al. [10] developed a biochar-supported nZVI composite for the remediation of Cr(VI)-contaminated soils. While decontamination improvements of the soil were achieved due to the hydrophobic nature of biochar, this feature makes it unfavorable for water-treatment applications when the contaminants of concern are ionized or non-hydrophobic in nature, such as heavy metal ions or chlorinated hydrocarbons. Therefore, to take advantage of the benefits of biochar for the removal of several groups of contaminants from the aqueous phase, surface functionalization needs to be considered.

A recent review of biochar-supported nZVI nanocomposites for water and soil decontamination published by Wang et al. (2019) has concluded that biochar significantly contributes to the removal of contaminants as it can attenuate contaminants on the surface of BC/nZVI and enhance electron transfer from nZVI to the target contaminants [11]. In a 2022 study by Islam et al. (2022), a fish-scale biochar nanocomposite with zinc oxide nanoparticles has been shown to be effective in treating Cr(VI)-contaminated wastewater, with maximum sorption capacity obtained at 90 mg/g [12]. Moreover, Fito et al. (2022) reviewed the effectiveness of various biochar-based nanocomposite photocatalysts, such as BC-TiO_2_ and BC-ZnO, for water and wastewater treatment and concluded that these nanocomposites have high activity, thermal stability, improved reusability, and a reduced recombination rate of the electron-hole pairs, which is highly favorable for photocatalytic applications [13].

In a previous study reported by Mortazavian et al. [2], a biochar sorbent produced from the pyrolysis of beetle-killed pine trees in the United States was modified through a simple heat treatment that transformed its surface from hydrophobic to hydrophilic. A subsequent impregnation with nZVI produced a biochar composite that was used for the removal of TCE from water [2]. Gao et al. [14] developed a zinc-biochar composite for Cr(VI) removal that resulted in up to 2.0 times higher removal efficiency than that of pristine biochar. Shang et al. [15] showed that nZVI supported on herb-residue biochar resulted in a 70% removal of Cr(VI) from water at pH = 4.0. Moreover, Qian et al. [16] supported nZVI on a silicon-rich biochar and showed a maximum of 30% Cr(VI) removal at pH= 4.0. Zhang et al. [17] developed a composite of biochar-supported nZVI stabilized by carboxymethyl cellulose for Cr(VI) removal from water and obtained around a 67% Cr(VI) removal efficiency at a pH of 4.0. 

In the present study, a commercial biochar with a naturally hydrophobic surface was first heat-treated under ambient air to make its surface hydrophilic and then impregnated with nZVI particles. The obtained composite, designated as HBC/nZVI, was then applied for the removal of Cr(VI) from simulated spiked contaminated water [2]. Surface characteristics alterations of biochar as a result of heat treatment, nZVI immobilization, and Cr(VI) sorption were investigated, and the Cr(VI) removal performance of pristine hydrophobic biochar (BC), heat-treated hydrophilic biochar (HBC), and heat-treated hydrophilic biochar impregnated with nZVI particles (HBC/nZVI) were compared. This is the first study that investigates surface characteristics and the Cr(VI) removal kinetics of a hydrophilic biochar with and without nZVI impregnation. 

## 2. Materials and Methods

### 2.1. Chemicals

Potassium dichromate (Cr_2_K_2_O_7_) as a source of chromium, iron (III) chloride hexahydrate (FeCl_3_·6H_2_O, 97% assay) as a source of zero-valent iron, and sodium hydroxide (NaOH, 97.0% assay) were purchased from Sigma Aldrich Corp. (St Louis, MO, USA). Sodium borohydride (NaBH_4_) was purchased from MP Biomedicals Corp. and sulfuric acid (H_2_SO_4_, >95% Assay) from J.T. Baker (Radnor, PA, USA). All the reagents were used as received. Biochar was provided by the Biochar Now company (Berthoud, CO, USA), with a particle size of 26–50 mesh. The biochar was produced from the pyrolysis of beetle-killed pine trees from national forests in the United States at a temperature of between 550 °C and 600 °C for 8 h, under a limited oxygen environment.

### 2.2. Synthesis Method

The as-received biochar (BC) was first subjected to heat treatment, aiming to transform its surface wettability from hydrophobic to hydrophilic, which is caused by alterations in its surface functional groups. This was accomplished by heating up biochar particles at 300 °C under ambient laboratory air (using a Thermo Scientific F48025 muffle furnace) for 24 h. This heat treatment generated hydrophilic biochar that was named HBC.

The impregnation of nZVI particles on HBC was accomplished following the method described in our previous study, in which the synthesis parameters have been optimized using a probe chemical [2]. Briefly, 125 mL of DI water was bubbled with argon gas for 15 min to expel its dissolved oxygen, then 1.89 g of HBC was added. A dose of 0.054 M of iron (III) chloride hexahydrate (FeCl_3_·6H_2_O) was added to the HBC mixture in water while the system was under magnetic stirring and bubbling with argon gas. After 15 min of agitation, the pH of the mixture was adjusted to 11.0 using a 0.1 M sodium hydroxide (NaOH) solution. Then, 50 mL of 0.054 M sodium borohydride (NaBH_4_) solution was added dropwise to the pH-adjusted mixture, along with ultrasonic irradiation, using a probe sonicator for 20 min (Qsonica Q500 0.5-inch diameter probe sonicator, frequency 20 kHz, 500 W power, 110 V, 2 s/2 s pulse/rest ratio, amplitude of 48 μm). When the addition of NaBH_4_ was completed, the system reacted for 2 h under constant argon bubbling. The particles were then separated from the mixture using vacuum filtration (Whatman qualitative filter, paper grade 4, pore size 20–25 mm), washed twice with low-oxygen DI water, and then vacuum-dried. This product was named HBC/nZVI. To prevent the oxidative passivation of nZVI in HBC/nZVI, the materials were kept under an argon atmosphere until the next applications and characterization.

### 2.3. Materials Characterization Methods

The surface morphologies of the three biochar sorbents (BC, HBC, and HBC/nZVI) were investigated using scanning electron microscopy (SEM; JSM-5610, JEOL Ltd., Tokyo, Japan), with 16 kV accelerating voltage and 20 mm working distance. Surface elemental analysis was conducted via energy dispersive X-ray spectroscopy (EDS; ISIS EDS, Oxford Instruments Company, Abingdon, UK) at 20 mm working distance. 

Surface functional groups of biochar samples before and after the reaction with Cr(VI) were investigated using Fourier transform infrared spectroscopy (FTIR) analysis through the attenuated total reflectance (ATR) technique (IRSpirit, Shimadzu Corp., Kyoto, Japan), in the wavenumber range of 400 to 4000 cm^−1^ and a scan number of 200. For the FTIR, samples were ground using a mortar and pestle and then mixed with KBr, with a mass ratio of 1:500 sample:KBr, and then pressed using a hydraulic press to form a transparent pellet. 

The zeta potentials of the biochar samples were measured using a Zetasizer (Malvern Zetasizer Ultra, Malvern Panalytical, PA, United States). For zeta potential measurement, biochar samples were first ground with a mortar and pestle and then sieved through sieve No. 325 (44 μm opening). Zeta potential suspensions were prepared by adding 0.005 g of powdered biochar in a 10 mM NaCl solution as the background medium, providing a concentration of 0.1%. The zeta potentials of all biochar samples were measured to be in the pH range of 3.0 to 9.0, adjusted using 0.1 M HCl and 0.1 M KOH solutions. The folded capillary cells (Malvern DTS 1080) were first washed with 5 mL Mili-Q water, followed by washing with 1 mL of sample, and were then filled with 700 μL of the sample for measurement. All zeta potential measurements were conducted at a temperature of 22 °C.

### 2.4. Cr(VI) Removal Experiments

Cr(VI) removal experiments were conducted in batch mode. A potassium dichromate stock solution of 1000 mg L^−1^ was prepared and acidified to pH < 2.0, using 37.5% concentrated hydrochloric acid, and kept refrigerated at 4 °C. Cr(VI) solutions with the desired concentrations were prepared in 100 mL volumetric flasks by diluting the prepared stock solution. The pH of the solutions was then adjusted to the predetermined values by using either diluted hydrochloric acid or sodium hydroxide. Predetermined quantities of sorbents (i.e., BC, HBC, and HBC/nZVI) were then added to the pH-adjusted Cr(VI) solutions. The testing glass bottles were sealed and placed on a rotary shaker at 40 rpm for specific reaction times. After the termination of the reaction time, the samples were filtered through 0.45 μm syringe filters to separate the sorbent particles from the aqueous solutions. The filtrate was then analyzed for Cr(VI) concentration using the colorimetric analysis method, based on the 1,5-diphenylcarbohydrazide method [18] (3500 Cr B and Hach Method 8023), using a DR/890 colorimeter (Hach, Iowa, United States; detection range 0.01–0.60 mg L^−1^).

### 2.5. Kinetic Models

The kinetics of Cr(VI) removal by using three various biochar adsorbents (i.e., BC, HBC, and HBC/nZVI) were investigated using pseudo-first- and pseudo-second-order models [4,19]. The pseudo-first-order model [20,21] and pseudo-second-order model [21,22] are expressed in Equations (1) and (2), respectively:

(1) $$q_{t}=q_{e}\left(1-e^{-k_{1}t}\right)$$

(2) qt=t1k2qe2+tqe

where $q_{t}$ and $q_{t}$ are the adsorption capacities (mg g^−1^), in terms of the mass of adsorbed adsorbate per unit mass of adsorbent, at equilibrium and time t (h), respectively, $k_{1}$ is the pseudo-first-order rate constant (h^−1^), and $k_{2}$ is the pseudo-second-order model constant (g mg^−1^ h^−1^).

To evaluate the kinetic models’ goodness-of-fit, $R^{2}$ alone is not enough. The normalized root mean square error (NRMSE) can better assess the degree of experimental data and fitted model agreement [21]:

(3) $$\mathrm{NRMSE}=\frac{\sqrt{\frac{\sum {\left(q_{t,\;exp}-q_{t,\;m}\right)}^{2}}{n}}}{\bar{q_{t,\;exp}}}$$

where $q_{t,\;exp}$ and $q_{t,\;m}$ are the experimental and calculated values (based on the model equation) of adsorbed Cr(VI) per unit mass of adsorbent (mg g^−1^), $\bar{q_{t,\;exp}}$ is the average of $q_{t,\;exp}$, and n is the number of data points.

## 3. Results

### 3.1. Sorbents Characterization

#### 3.1.1. Surface Morphology

**Figure 1:**
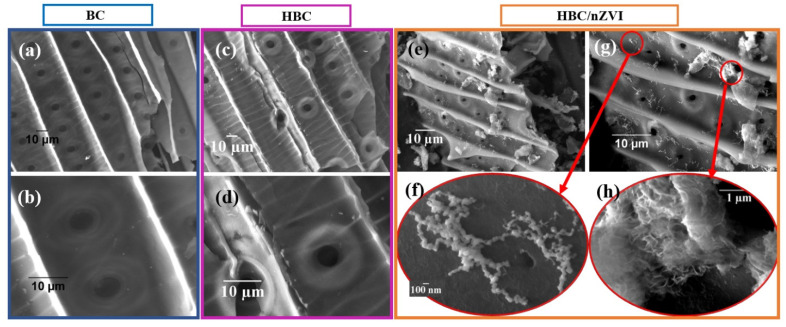
Surface morphology of (**a**,**b**) virgin BC, (**c**,**d**) HBC, and (**e**–**h**) HBC/nZVI.

**Figure 2:**
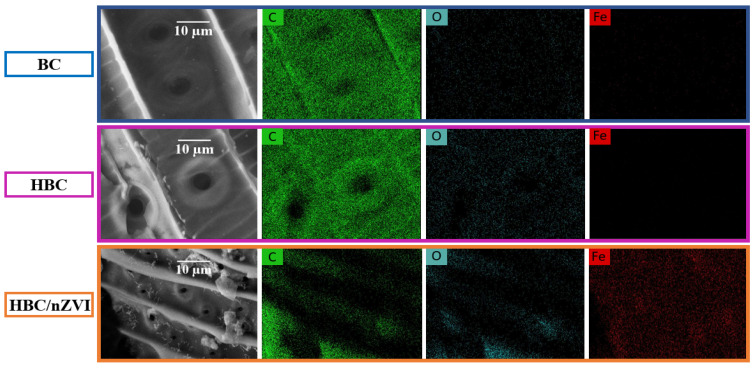
Elemental maps from the EDS analysis of BC (**first row**), HBC (**middle row**), and HBC/nZVI (**bottom row**), assigning green to carbon, cyan to oxygen, and red to iron.

The surface morphology and surface elemental composition of virgin biochar (BC), hydrophilic biochar (HBC), and HBC impregnated with nZVI (HBC/nZVI) were investigated using SEM and EDS mapping, as shown in Figure 1 and Figure 2, respectively. Figure 1a,b show that the virgin BC had a smooth surface and consisted of parallel tubes, with features having spherical holes of approximately 3 μm in diameter. All spherical holes were equally positioned and spaced at distances of 10 to 15 µm. The presence of these features allows for an adequate incubation and flow of the contaminants. Heat-treated hydrophilic BC (HBC), as shown in Figure 1c,d, had the same tube-and-hole structure as virgin BC, while the surface was not as smooth as virgin BC and contained several cracks. The cracks could potentially be beneficial for contaminant uptake as they increase the surface area. It should be noted that these cracks, while visible throughout the structure, were not open and the mechanical stability of HBC was still intact. The surface of HBC/nZVI in Figure 1e,g showed two additional features on the surface: (i) nZVI particles with a spherical shape and an average diameter of 50 nm, magnified in Figure 1f, and (ii) iron oxide with a flaky texture, magnified in Figure 1h. For more information about the surface chemistry and X-ray photoelectron spectroscopy (XPS) results of these three types of biochars, please refer to our previously published work [2].

Figure 2 illustrates the elemental maps of BC, HBC, and HBC/nZVI that resulted from EDS analysis, assigning a green color to carbon, cyan to oxygen, and red to iron. The first row in Figure 2 corresponds to BC and showed that the BC mainly consisted of a carbon element, with a negligible quantity of oxygen, and no iron. In the second row of Figure 2, HBC is shown to consist of carbon and a higher quantity of oxygen compared to BC; this has previously been shown to be due to the formation of carbonyl functional groups during the heat treatment, which is responsible for the increased hydrophilicity [2]. No iron element was detected in the HBC, as expected. The third row in Figure 2 shows that HBC/nZVI had the greatest amount of oxygen among the three forms of biochar sorbents, stemming from the induced oxygen-containing functional groups as a result of the heat treatment, as well as from iron oxide as a result of the nZVI surface oxidation forming the well-known nZVI core-shell structure [19].

#### 3.1.2. Fourier Transform Infrared Spectroscopy (FTIR) Analysis

**Figure 3:**
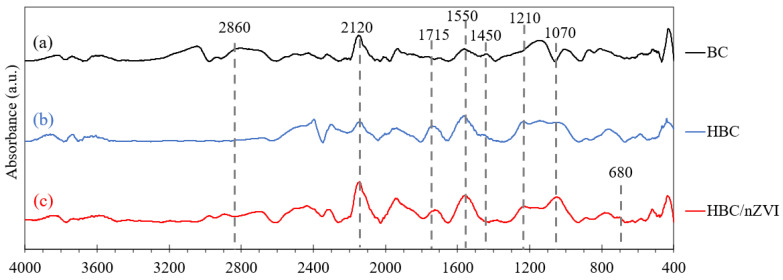
FTIR spectra of BC, HBC, and HBC/nZVI (before contact with the Cr(VI) solution).

FTIR spectrums of BC, HBC, and HBC/nZVI (before reacting with Cr(VI) solutions) were obtained in the wavenumber range of 400 to 4000 cm^−1^, using the ATR technique. The results are shown in Figure 3.

The peaks at 1070 and 1210 cm^−1^ can be assigned to the C-O-C and C-O functional groups [2,23,24], and the peak at 1715 cm^−1^ can be assigned to the carbonyl (C=O) group [25], all of which are oxygen-containing hydrophilic groups with enhancing effects on the wettability of porous carbons in aqueous solutions [26]. As observed in Figure 3, these three additional peaks were not observed in the FTIR spectrum of raw BC (Figure 3, line a), while in the HBC and HBC/nZVI spectra (Figure 3, lines b and c), the peaks from C-O-C, C-O and C=O were detected. The peak at 1550 cm^−1^ can be assigned to the asymmetric stretching vibrations of COO¯ [27], inducing hydrophilicity. In Figure 3, it can be observed that the intensity of this peak was enhanced in HBC (Figure 3, line b) and HBC/nZVI (Figure 3, line c), compared to the raw BC (Figure 3 line a), indicating the enhanced hydrophilicity of the two modified biochars (i.e., HBC and HBC/nZVI) compared to the unmodified BC. This demonstrates that the heat treatment at 300 °C under ambient air induced the formation of hydrophilic oxygen-containing functional groups on the surface of the biochar, presumably due to the increased likelihood of oxygen interaction with the biochar’s surface at higher temperatures. On the other hand, in all three spectra shown in Figure 3, peaks are clearly visible at 2860 and 1450 cm^−1^ that can be attributed to C-H stretching and bending vibrations [24,27]. As seen in Figure 3, the intensity of the C-H peaks decreased from raw BC (Figure 3, line a) to HBC (Figure 3, line b) and HBC/nZVI (Figure 3, line c), indicating the diminished hydrophobicity of biochar after heat treatment and nZVI impregnation.

The existence of a bond between Iron and biochar in the HBC/nZVI composite was corroborated by the existence of an Fe-O-H peak at 680 cm^−1^ [28] that can only be observed in the HBC/nZVI FTIR spectrum. Finally, the peaks at 2120 cm^−1^ that can be observed in the FTIR spectra of BC, HBC, and HBC/nZVI corresponded to the C≡C bonds in the biochars [28].

#### 3.1.3. Zeta Potential Studies

**Figure 4:**
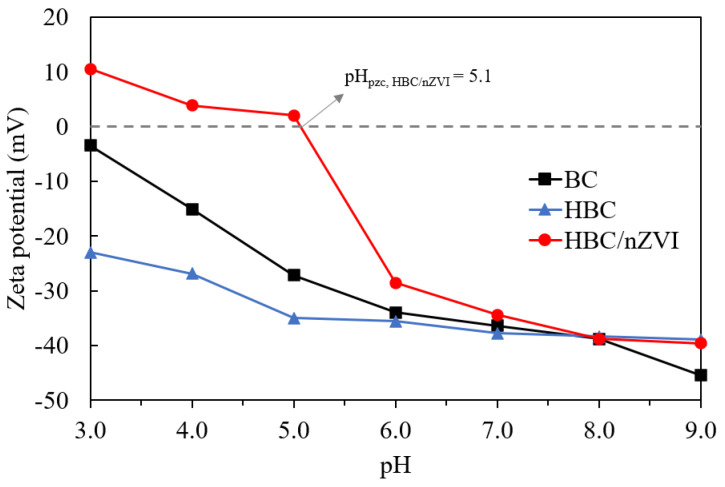
Zeta potential data of BC, HBC, and HBC/nZVI as a function of pH (measured in 10 mM NaCl electrolyte solution at 25 °C).

The zeta potential analysis of BC, HBC, and HBC/nZVI was measured in an environmentally relevant pH range of 3.0 to 9.0 (Figure 4), with a refractive index set at 2.7, which is the reported refractive index for graphite [29]. The results are shown in Figure 4.

As seen in Figure 4, the zeta potential of all three biochar sorbents decreased with increasing pH. This is not surprising; at higher pH values, the acidic protons associate with the double layer, thus making the zeta potential more positive. This decreasing trend was steeper for BC than for HBC, in such a way that although HBC showed a more negative surface charge than BC up to the pH of 8.0, at a highly basic pH of 9.0, BC showed a more negative surface. The HBC/nZVI composite, on the other hand, had a different zeta potential pattern than the two non-impregnated biochars; it showed both positive and negative zeta potential values in the examined pH range. Based on the results, the point of a zero charge of HBC/nZVI was determined to be pH_pzc_, HBC/nZVI = 5.1, below which the surface of HBC/nZVI is positively charged, and, in pH values greater than that, the surface charge is negative. The differences between non-impregnated BC and HBC with the HBC/nZVI composite, in terms of the surface charge alterations as a function of pH, possibly stemmed from the presence of iron in the composite, affecting the surface charge characteristics.

### 3.2. Cr(VI) Removal 

#### 3.2.1. Effects of Solution pH on Cr(VI) Removal Performance

**Figure 5:**
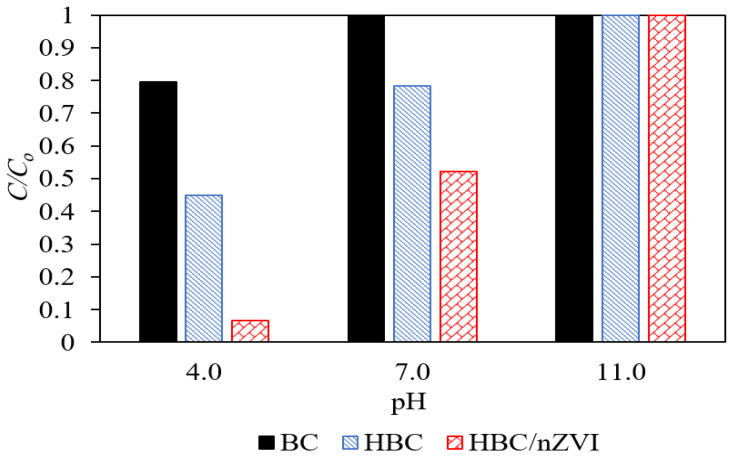
Effects of solution pH on the Cr(VI) removal performance of BC (solid black columns), HBC (blue dashed columns), and HBC/nZVI (red plaid columns). Experimental conditions: Initial Cr(V) concentration = 10 mg L^−1^, reaction time = 24 h, adsorbent dose = 1.5 g L^−1^.

Figure 5 shows the effects of solution pH on the effectiveness of BC, HBC, and HBC/nZVI, as examined at three pH levels of 4.0 (acidic), 7.0 (neutral), and 11.0 (basic), with an initial Cr(VI) concentration of 10 mg L^−1^, a reaction time of 24 h, and an adsorbent dose of 1.5 g L^−1^.

As shown in Figure 5, an increasing pH results in the decreased Cr(VI) removal efficiency of BC, HBC, and HBC/nZVI. This was demonstrated by an increased C/C_0_ ratio (i.e., the ratio of Cr(VI) concentration in the treated solutions to the initial Cr(VI) concentration) with increasing pH. All three sorbents showed almost no Cr(VI) removal in a highly basic pH of 11.0. At the pH of 4.0 and 7.0, BC showed the lowest removal efficiency, and HBC/nZVI showed the highest removal efficiency.

The effects of pH on Cr(VI) removal efficiency stems from (a) changes in chromium speciation, as well as (b) variations of the adsorbents’ surface charge. Chromate (CrO_4_^2−^), bichromate (HCrO_4_^−^), and dichromate (Cr_2_O_7_^2−^) are three possible forms of Cr(VI) in aqueous solutions. At pH values of between 1.0 and 6.0 (i.e., acidic medium), bichromate is the dominant form of Cr(VI), while at pH values above 6.0, chromate dominates [30,31]. When using BC and HBC with no embedded nZVI particles, the electrostatic attraction/repulsion plays a major role in Cr(VI) adsorption efficiency. Based on the results from the zeta potential analysis (Figure 4), at a pH of 4.0, the surface of BC and HBC are negatively charged, repulsing all Cr(VI) anions. However, a pH of 4.0 was favorable for the removal of contaminants. For example, a pH higher than 4 induced a positive surface charge on HBC/nZVI as it was lower than pH_pzc, HBC/nZVI_ = 5.1. Figure 5 shows that at pH = 4.0, HBC/nZVI yielded a significantly greater removal efficiency of 93.4%, compared to BC and HBC, which yielded 55% and 20.5% removal. Although the zeta potential results in Figure 4 show that HBC had a more negatively charged surface at a pH of 4.0 compared to BC (ZP_BC_= −15.1 mV and ZP_HBC_ = −26.9 mV), the hydrophilicity of HBC was probably the reason for its greater Cr(VI) removal performance than hydrophobic BC. At a pH = 7.0, although the zeta potentials of BC and HBC were closely similar (ZP_BC_= −36.4 mV and ZP_HBC_ = −37.8 mV), HBC showed 21.7% Cr(VI) removal, while BC showed almost zero removal. This again confirmed that despite a stronger negative surface charge for HBC in most pH values compared to BC, HBC removed Cr(VI) anions more efficiently than BC, presumably due to its hydrophilicity. At a pH of 11.0, all three biochar sorbents showed nearly zero Cr(VI) removal, which is consistent with their highly negative surfaces in basic pH environments.

When using HBC/nZVI, Cr(VI) removal occurs through simultaneous adsorption and chemical reduction via electron donation from Fe^0^ to Cr(VI) anions. As shown in Equations (4)–(7) below, it is believed that increasing the hydrogen ion concentration in the aqueous solution facilitates Cr(VI) reduction to Cr(III) [32]. This mechanism confirms the experimental observations shown in Figure 5, indicating significantly enhanced Cr(VI) removal in acidic pH environments when using HBC/nZVI. These results are in agreement with the previous studies on the application of nZVI for Cr(VI) reduction [19,32,33]. In addition, based on Equations (8) and (9), higher pH values are favorable for the precipitation of Fe(III)/Cr(III) complexes, resulting in nZVI passivation and, consequently, in diminished Cr(VI) removal efficiency [32].

(4) $$2{\mathrm{HCrO}}_{4}^{-}+3{\mathrm{Fe}}^{0}+14H^{+}\;\to \;2{\mathrm{Cr}}^{3+}+3{\mathrm{Fe}}^{2+}+8H_{2}O$$

(5) $$2{\mathrm{CrO}}_{4}^{2-}+3{\mathrm{Fe}}^{0}+16H^{+}\;\to \;2{\mathrm{Cr}}^{3+}+3{\mathrm{Fe}}^{2+}+8H_{2}O$$

(6) $${\mathrm{HCrO}}_{4}^{-}+3{\mathrm{Fe}}^{2+}+7H^{+}\;\to \;2{\mathrm{Cr}}^{3+}+3{\mathrm{Fe}}^{3+}+4H_{2}O$$

(7) $$2{\mathrm{CrO}}_{4}^{2-}+3{\mathrm{Fe}}^{2+}+8H^{+}\;\to \;2{\mathrm{Cr}}^{3+}+3{\mathrm{Fe}}^{3+}+4H_{2}O$$

(1 − n)Fe^3+^ + nCr^3+^ + 3H_2_O → Cr_n_Fe_1−n_(OH)_3_ + 3H^+^ (8)

(1 − n)Fe^3+^ + nCr^3+^ + 2H_2_O → Cr_n_Fe_1−n_OOH + 3H^+^ (9)

The results from pH studies illustrated that although hydrophilicity and chemical reduction significantly enhanced the Cr(VI) removal performance of biochar, strong electrostatic repulsion can diminish the effectiveness as the Cr(VI) anions’ contact with sorbents would not be favorable.

#### 3.2.2. Cr(VI) Removal Efficiency Comparison between the BC, HBC, and HBC/nZVI Sorbents 

**Figure 6:**
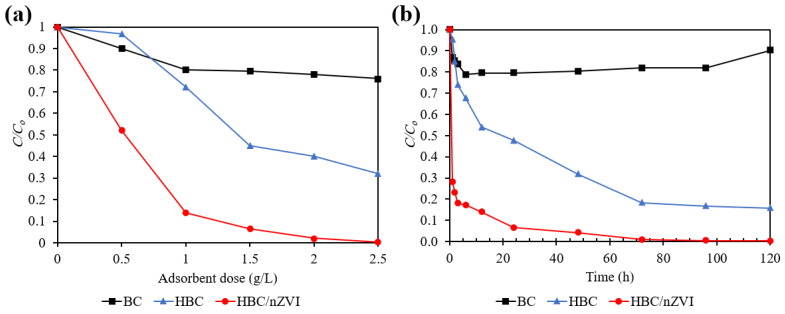
Effects of (**a**) adsorbent dose (initial Cr(V) concentration = 10 mg L^−1^, pH = 4.0, reaction time = 24 h) and (**b**) reaction time (initial Cr(V) concentration = 10 mg L^−1^, pH = 4.0, adsorbent dose = 1.5 g L^−1^) on the Cr(VI) removal efficiency using BC, HBC, and HBC/nZVI.

The effectiveness of virgin biochar (BC), hydrophilic biochar (HBC), and HBC impregnated with nZVI (HBC/nZVI) for Cr(VI) removal was investigated in batch mode. Figure 6 shows the results.

Under constant experimental conditions with a pH of 4.0, an initial Cr(VI) concentration of 10 mg L^−1^, and a reaction time of 24 h, Figure 6a shows that as the adsorbent dose increased, the removal efficiency of all three examined sorbents (i.e., BC, HBC, and HBC/nZVI) led to enhanced Cr(VI) removal capacity. However, among the three biochar materials tested, HBC/nZVI was the most efficient sorbent in terms of Cr(VI) removal performance. For example, an increased dose from 0.5 g L^−1^ to 2.5 g L^−1^ enhanced the Cr(VI) removal percentage from 48% to 100% for HBC/nZVI, from 3% to 68% for HBC, and from 10% to 24% for BC. The reduced removal efficiency of BC based on an increasing dose for the removal of a constant number of Cr(VI) ions from the solution can be attributed to the hydrophobicity of BC particles. It is believed that the high hydrophobic properties of the virgin BC prevented a uniform and effective mixing process with the aqueous contaminant matrix. Additionally, the clustering of BC particles was recorded as the result of an increased dose of these particles in the solution, preventing and limiting their efficient interaction with aqueous Cr(VI) ion solutions. 

However, as depicted in Figure 6a, a simple heat treatment on raw BC under ambient air transformed its surface wettability properties. Upon heat treatment, HBC demonstrated a significantly superior response for Cr(VI) removal than virgin BC. This is presumed to be mainly due to the enhanced interaction between the adsorbate ions and adsorbent surface, as a result of HBC’s favorable hydrophilicity. Although the effectiveness of HBC/nZVI for Cr(VI) removal is greater than HBC, the trend of the enhanced performance of HBC and HBC/nZVI as a result of increased adsorbent doses is similar (Figure 6a). This highlights that the heat treatment procedure could resolve the weak interaction between the biochar’s surface and Cr(VI) ions as it changed the attenuating removal performance observed for raw BC.

Figure 6b shows the effect of reaction time (1, 2, 3, 6, 12, 24, 48, 72, 96, and 120 h) on the Cr(VI) removal performances of BC, HBC, and HBC/nZVI, at a pH of 4.0, an initial Cr(VI) concentration of 10 mg L^−1^, and an adsorbent dose of 1.5 g L^−1^. It was observed that by increasing the reaction time from 1 h to 120 h, the continued removal of Cr(VI) from the solution occurred by using HBC and HBC/nZVI. However, BC did not follow the same trend and Cr(VI) desorption was initiated after 6 h of reaction. This demonstrates that the interaction between Cr(VI) anions and the BC surface is based on a weak physisorption interaction. 

The greatest removal percentage for BC was 21.3%, which occurred after 6 h of reaction, after which desorption was initiated, leading to a 9.8% removal after 120 h. After 6 h of reaction, HBC showed a 32.6% removal efficiency, which is 1.5 times greater than that of BC, while HBC/nZVI showed an 82.8% removal efficiency, which is 3.9 times greater than that of BC.

Regarding equilibrium conditions, the removal percentage for HBC started to plateau after 72 h at 81.7%, reaching 84.1% after 120 h of reaction. HBC/nZVI started to plateau after 72 h at 99.2% removal and reached 99.7% removal after 120 h.

In order to better compare the removal rates and capacities of the three examined biochar sorbents, the kinetics of the reactions were investigated; the results will be presented in the next section.

#### 3.2.3. Kinetics of Cr(VI) Removal Using BC, HBC, and HBC/nZVI

**Figure 7:**
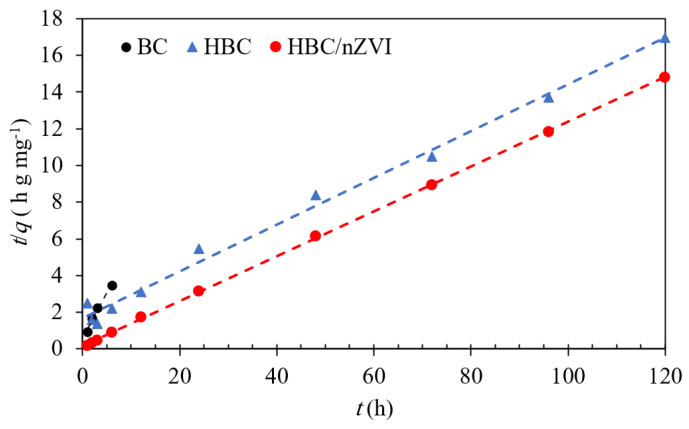
Pseudo-second-order kinetic models for aqueous Cr(VI) removal using BC, HBC, and HBC/nZVI. Dashed lines represent fitted pseudo-second-order models, and the markers represent experimental data points. Experimental conditions: initial Cr(V) concentration = 10 mg L^−1^, pH = 4.0, adsorbent dose = 1.5 g L^−1^.

The kinetics of Cr(VI) removal using BC, HBC, and HBC/nZVI were examined by fitting the experimental data points to the pseudo-first order, pseudo-second order, and the Elovich kinetic models. All three sorbents showed the best fit to the pseudo-second-order model (Figure 7). Table 1 presents the models’ characteristics. It should be noted that for BC, only the data points in the adsorption range were considered, while the points after the initiation of desorption (i.e., after 6 h) were excluded.

For BC, HBC, and HBC/nZVI, the high values of $R^{2}$ and small NRMSE values determined a good fit of the experimental data with the fitted models.

As observed in Table 1, BC showed the greatest Cr(VI) removal rate among the three various biochar sorbents. However, while highly effective within a 6h period, BC ceased adsorbing Cr(VI) after 6 h of reaction and started desorption (Figure 6). It should be noted that only the data points in the adsorption stage of BC were used in the kinetic model investigations. The adsorption capacity predicted by the pseudo-second-order model (i.e., $q_{e}$) was 2.05 mg of adsorbed Cr(VI) per gram of BC. The hydrophilic BC, known as HBC, showed a slower Cr(VI) uptake rate compared to BC, while its ultimate calculated capacity was 3.8 times greater than that of BC. After impregnating HBC with nZVI, the HBC/nZVI composite increased the Cr(VI) removal rate by a factor of 10 compared to HBC and showed a greater $q_{e}$ of 8.18 mg g^−1^. 

The results of the kinetic studies indicated that the simple heat treatment applied on raw BC (300 °C for 24 h) had the most significant effect on enhancing the biochar’s Cr(VI) removal capability. We postulate that it was due to the transformation of the biochar’s wettability characteristics, based on the surface functional group transformation [2], mainly the formation of carbonyl groups. In comparison with previously studied similar adsorbents for aqueous Cr(VI) removal, the heat-treated hydrophilic biochar introduced in the present study showed favorable removal capacity. Table 2 summarizes the results of similar published studies on the application of biochar and modified biochar for the removal of Cr(VI) from the aqueous phase. For example, Agrafioti et al. [34,35] applied two types of biochar produced from the pyrolysis of rice husk and the organic fraction of municipal solid waste in their pristine form [34] and impregnated with Fe^0^ and Fe^3+^ [35], employing them for aqueous Cr(VI) removal. Their results showed that the impregnation of rice husk biochar with Fe^0^ did not significantly improve its Cr(VI) sorption capacity and that the impregnation of municipal solid waste biochar with Fe^0^ had a negative effect on its Cr(VI) removal performance [35]. However, Fe^3+^ impregnation improved the Cr(VI) removal efficiency of both biochar types (Table 2) [35].

For comparison with the activated carbon, which is a widely used adsorbent in the water treatment industry, Mortazavian et al. [19] investigated aqueous Cr(VI) removal, using Filtrasorb-400 granular activated carbon (Calgon Carbon Corp., Pittsburgh, PA, United States) in its pristine form (AC) and again after impregnation with nZVI (AC/nZVI). They obtained Cr(VI) adsorption capacities of 4.79 and 6.67 mg g^−1^ using AC and AC/nZVI, respectively, as predicted by the pseudo-second-order model [19].

A comparison of the results from the present study with the previously reported biochar sorbents for aqueous Cr(VI) removal (Table 2) indicates the significant advantages to the introduced heat treatment method. This simple, cost-effective step, which does not involve any harsh chemical treatment for modification of the biochar, is highly efficient for aqueous Cr(VI) removal. The HBC/nZVI composite also demonstrated an excellent removal capacity compared to the previously reported, chemically modified biochars.

The results of this study suggest that biochar, as an inexpensive adsorbent produced from forestry or crop residues, can replace expensive alternatives such as activated carbon, which is currently widely used for the removal of heavy metals from water. Ultimately, the heat-treated biochar (HBC) itself can be considered as an efficient sorbent for aqueous Cr(VI) remediation. It also can be considered as an economical substitute for porous materials, such as mesoporous silicas [43,44] and porous carbons [19,45,46], for the immobilization of nZVI and other zero-valent metals.

In addition, based on a previous study by Mortazavian et al. [19] that showed high regeneration and reusability for an activated carbon/nZVI nanocomposite for Cr(VI) removal from water, it is anticipated that the HBC/nZVI composite shows similar performance in terms of reusability efficiency, as both these composites contain a carbonaceous adsorbent that is impregnated with nZVI. This assumption can be further investigated in future studies.

### 3.3. FTIR Analysis of Sorbents after Reacting with Cr(VI)

**Figure 8:**
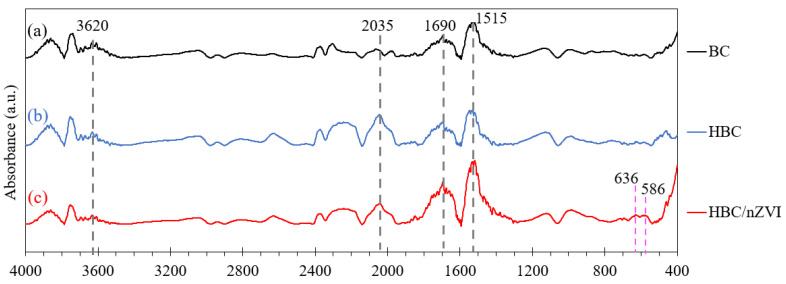
FTIR spectra of BC, HBC, and HBC/nZVI after reacting with Cr(VI) aqueous solutions.

After using BC, HBC, HBC/nZVI for Cr(VI) removal, the used particles were analyzed for their functional groups using FTIR. The results are shown in Figure 8.

In Figure 8, the peak at 3600 cm^−1^, which was observed in all FTIR spectra of the BC, HBC, and HBC/nZVI used, can be attributed to the hydroxyl group [28] that presumably originated from the remaining moisture in the sorbents after being used in the aqueous solutions for Cr(VI) removal. This is consistent with the results from Gan et al., analyzing the FTIR of zinc-biochar nanocomposite after application for Cr(VI) adsorption, showing the hydroxyl groups formed on the biochar composite after adsorption. As shown in Figure 8, the peaks at 586 cm^−1^ and 636 cm^−1^ were observed only in the HBC/nZVI spectrum and can be attributed to chromium (III) oxide (Cr_2_O_3_) [47]. This demonstrated that the chemical reduction of Cr(VI) to Cr(III) only occurred in HBC/nZVI, presumably due to the presence of nZVI particles (Equations (4)–(7)).

A comparison of the FTIR spectra of the three biochar sorbents, both before being used for Cr(VI) adsorption (Figure 3) and afterward (Figure 8), shows that after Cr(VI) adsorption, the peaks associated with C=O and COO¯ were shifted to lower wavenumbers (from 1715 to 1690 cm^−1^ and from 1550 to 1515 cm^−1^, respectively). This is consistent with previous, similar research reports on Cr(VI) sorption onto biochar and biochar composites [14], demonstrating that the carbonyl and carboxyl groups are the main functional groups involved in Cr(VI) sorption onto biochar. Lastly, Figure 8 also shows that the peaks at 2035 cm^−1^, attributable to the C≡C group, were present on BC, HBC, and HBC/nZVI after Cr(VI) adsorption.

## 4. Conclusions

This study introduced an inexpensive and environmentally friendly sorbent for the efficient removal of heavy metals, specifically, hexavalent chromium species, from aqueous solutions. The present study first treated the surface functional groups of biochar (produced from pine tree residues) with a simple heat treatment at 300 °C under ambient air, then impregnated it with nZVI particles. The obtained composite, designated as HBC/nZVI, showed a 93.4% Cr(VI) removal efficiency at pH = 4.0, which is significantly higher than the reported efficiencies in similar previous studies. The results demonstrated that the application of simple heat treatment at 300 °C for 24 h (producing HBC) significantly enhanced the biochar’s Cr(VI) adsorption capacity, as it increased its Cr(VI) sorption capacity from 2.05 mg g^−1^ to 7.87 mg g^−1^. Impregnating HBC with nZVI further increased the Cr(VI) removal capacity to 8.18 mg g^−1^. FTIR studies demonstrated that oxygen-containing functional groups formed after heat treatment, which was the underlying cause for the observed enhanced sorption capacity of HBC compared to BC. The impregnation of nZVI particles on HBC, producing the HBC/nZVI composite, further enhanced the Cr(VI) removal capacity and boosted its Cr(VI) removal rate, through the addition of a chemical reduction mechanism to physical adsorption. Zeta potential analysis showed that BC and HBC had a negative surface charge from 3.0 < pH < 9.0, while the point-of-zero-charge of HBC/nZVI was determined at 5.1. Evaluating the effects of pH on the Cr(VI) removal efficiencies of BC, HBC, and HBC/nZVI performed favorably in acidic media for all three sorbents. HBC/nZVI showed the greatest removal rate at a pH of 4.0 and 7.0. At a pH of 11.0, all three investigated sorbents showed almost no removal capacity of Cr(VI), which can be attributed to their highly negative surface charges in the basic medium, as demonstrated from zeta potential measurement analysis, repulsing the Cr(VI) anion species.

The results of this study suggest that heat-treated biochar (i.e., HBC) could be the preferred sorbent for the in situ remediation of Cr(VI)-contaminated groundwaters. This is an inexpensive and highly efficient adsorbent that is active for extended contact times and that significantly minimizes contaminant treatment costs. In addition, when a faster adsorption process is desired, the HBC/nZVI nanocomposite could be considered the preferred option as it provides a significantly higher removal rate at a faster pace. HBC also provides a cost-effective support medium for nZVI immobilization. This study has introduced, for the first time, a biochar-based nanocomposite for the Cr(VI) decontamination of water that is also economically preferable to the commonly used supporting media, such as activated carbon, carbon nanotubes, or mesoporous silica, as previously introduced by several researchers for nZVI immobilization for water treatment applications.

The results of this study can benefit the environmental engineering community by introducing a highly efficient nanocomposite for water treatment that is made from forestry residues, helping to preserve limited freshwater resources that have been contaminated due to industrial activities.

## Figures and Tables

**Table 1:** Pseudo-second-order kinetic model characteristics for the removal of Cr(VI) using BC, HBC, and HBC/nZVI under an initial Cr(V) concentration = 10 mg L^−1^, pH = 4.0, adsorbent dose = 1.5 g L^−1^.

	k (g mg^−1^ h^−1^)	q_e_ (mg g^−1^)	R^2^	NRMSE
BC	0.39	2.05	0.98	0.08
HBC	0.01	7.87	0.99	0.09
HBC/nZVI	0.10	8.18	>0.99	0.11
Pseudo-second order kinetic model: qt=t1k2qe2+tqe (Equation (2))				

**Table 2:** Summary of previous studies on the application of biochar for aqueous Cr(VI) removal.

Feedstock Materials	Modification Technique	pH of Cr(VI) Solution	Initial Cr(VI) Concentration (mg L^−1^)	Adsorbent Dose (g L^−1^)	Cr(VI) Removal Efficiency (%)	Ref. No.
**Pristine Biochar**						
Rice Husk	None	7 to 9.5	0.19	16	18%	[34]
Municipal solid waste	None	7 to 9.5	0.19	16	44%	[34]
Sugar beet tailing	None	4.0	100	2	55%	[36]
Oak wood	None	4.0	10	10	19%	[37]
Oak bark	None	4.0	10	10	10%	[37]
Municipal sewage sludge	None	5.0	50	2	10%	[38]
Pomelo peel	None	4.0	200	1	5%	[39]
Pine tree residues	None	4.0	10	1.5	20.5%	This study
**Modified biochar**						
Rice husk	Fe^0^ impregnation	7.0	0.85	16	24%	[35]
Rice husk	Fe^3+^ impregnation	7.0	0.85	1	35%	[35]
Municipal solid waste	Fe^0^ impregnation	7.0	0.85	16	14%	[35]
Municipal solid waste	Fe^3+^ impregnation	7.0	0.85	1	89%	[35]
Rice husk	KOH and Polyethylenimine surface treatments	4.0	100	1	98%	[40]
Pisum sativum	Encapsulation of starch hydrogel	4.0	50	2	70%	[41]
Pomelo peel	K_2_FeO_4_-promoted Pyrolysis	4.0	200	1	8%	[39]
Wheat straw	carboxymethyl cellulose stabilization and FeS deposition	4.0	100	0.72	85%	[42]
Pine tree residues	Heat treatment at 300 °C	4.0	10	1.5	55%	This study
Pine tree residues	Heat treatment and Fe^0^ impregnation	4.0	10	1.5	93%	This study

## Data Availability

Not applicable.
